# Interactions between Skeletal Muscle Myoblasts and their Extracellular Matrix Revealed by a Serum Free Culture System

**DOI:** 10.1371/journal.pone.0127675

**Published:** 2015-06-01

**Authors:** Vishal Chaturvedi, Danielle E. Dye, Beverley F. Kinnear, Toin H. van Kuppevelt, Miranda D. Grounds, Deirdre R. Coombe

**Affiliations:** 1 School of Biomedical Science, CHIRI Biosciences Research Precinct, Faculty of Health Sciences, Curtin University, Perth, Western Australia, Australia; 2 Radboud Institute of Molecular Life Sciences, Radboud University Medical Center, Nijmegen, The Netherlands; 3 School of Anatomy, Physiology and Human Biology, University of Western Australia, Perth, Western Australia, Australia; University of California, San Diego, UNITED STATES

## Abstract

Decellularisation of skeletal muscle provides a system to study the interactions of myoblasts with muscle extracellular matrix (ECM). This study describes the efficient decellularisation of quadriceps muscle with the retention of matrix components and the use of this matrix for myoblast proliferation and differentiation under serum free culture conditions. Three decellularisation approaches were examined; the most effective was phospholipase A2 treatment, which removed cellular material while maximizing the retention of ECM components. Decellularised muscle matrices were then solubilized and used as substrates for C2C12 mouse myoblast serum free cultures. The muscle matrix supported myoblast proliferation and differentiation equally as well as collagen and fibronectin. Immunofluorescence analyses revealed that myoblasts seeded on muscle matrix and fibronectin differentiated to form long, well-aligned myotubes, while myoblasts seeded on collagen were less organized. qPCR analyses showed a time dependent increase in genes involved in skeletal muscle differentiation and suggested that muscle-derived matrix may stimulate an increased rate of differentiation compared to collagen and fibronectin. Decellularized whole muscle three-dimensional scaffolds also supported cell adhesion and spreading, with myoblasts aligning along specific tracts of matrix proteins within the scaffolds. Thus, under serum free conditions, intact acellular muscle matrices provided cues to direct myoblast adhesion and migration. In addition, myoblasts were shown to rapidly secrete and organise their own matrix glycoproteins to create a localized ECM microenvironment. This serum free culture system has revealed that the correct muscle ECM facilitates more rapid cell organisation and differentiation than single matrix glycoprotein substrates.

## Introduction

Skeletal muscle tissue comprises 40–50% of the human body mass and is essential for body movement, metabolism and thermoregulation. Although muscle has excellent regenerative capacity, when an injury results in a significant loss of muscle, natural repair processes are unable to bridge the gap between the remaining segments of the skeletal muscle fibres, leading to loss of muscle tissue and functional deficit. One approach for treating volumetric muscle loss is tissue engineering, where the use of biological scaffolds composed of extracellular matrix (ECM) derived from animal tissue is being explored. Natural ECM, secreted by resident tissue cells, should provide the optimal physical, chemical and biological cues to support regeneration of that tissue. Scaffolds of native ECM have been used to regenerate heart [1] and liver [2] *ex vivo* and decellularized porcine small intestinal submucosa (SIS) ECM has been used in human and animal models to reconstruct skin [3, 4], urinary bladder [5], abdominal wall defects [6, 7], rotator cuff tendon [8], and load bearing skeletal muscle [9].

Decellularized skeletal muscle is an ideal source of muscle specific ECM and provides a system to study the role of ECM in muscle regeneration. Skeletal muscle has three layers of ECM: the endomysium surrounds individual fibres and is in intimate contact with myofibres and satellite cells, while the perimysium, and epimysium surround groups of myofibres and the entire muscle, respectively. The ECM provides both structural support and biochemical cues that direct muscle formation. Collagens I and III are the major structural proteins in skeletal muscle ECM, whereas collagen VI is an essential component of the satellite cell niche and contributes to the regulation of satellite cell self-renewal [10]. Laminins containing the α2 chain are located in the skeletal muscle basement membrane and are required for myotube formation and the prevention of apoptosis [11, 12]. Fibronectin binds collagen and laminin and contributes to myoblast adhesion, migration and differentiation [13], as well as being involved in satellite cell expansion via Wnt7a signaling [14]. In addition, proteoglycans, such as decorin and perlecan, can interact with a number of different ligands via their core proteins and their glycosaminoglycan (GAG) chains, and as a consequence have diverse functions in skeletal muscle [15]. Specifically, both perlecan and decorin are involved in myostatin signaling, and whereas perlecan contributes to the maintenance of fast muscle fibres, decorin is involved in the proliferation and differentiation of myogenic cells [16, 17]. The GAG chains of proteoglycans also contribute to myogenesis by sequestering growth factors like fibroblast growth factor 2 (FGF-2) and hepatocyte growth factor (HGF); both of which stimulate skeletal muscle cell proliferation and inhibit differentiation [15]. Thus, a decellularised muscle matrix will ideally retain collagens, laminin, fibronectin and proteoglycans with their GAG chains.

A number of different decellularisation techniques have been investigated for muscle tissue. Stern et al (2009) used trypsin and Triton X-100 to decellularize rat muscle. This approach efficiently removed nuclear material and retained collagen but resulted in a loss of GAGs [18]. DeQuach et al (2010) used 1% sodium dodecyl sulphate (SDS) as decellularizing agent, and demonstrated good retention of a range of matrix proteins and GAGs [19]. Recently, Gillies et al (2011) described a method for decellularizing skeletal muscle without detergents or proteolytic enzymes, which also showed good retention of a range of matrix components including GAGs [20].

Functional analyses of scaffolds in tissue culture traditionally use fetal bovine and horse sera as a source of growth factors. However, sera contain a diversity of ill-defined components, which makes it difficult to assess the contribution of specific ECM molecules contained within decellularized tissue scaffolds. A limited number of serum free culture systems for skeletal muscle cells have been developed by others, but most contain an undefined serum substitute [21]. In one case hydrophilic substrates of 3-trimethoxysilyl propyl-diethylenetriamine were used to promote attachment of primary dissociated skeletal muscle cells, even though the ECM proteins vitronectin, laminin and tenascin-C were included in the media [22, 23].

Here we describe a method for decellularizing skeletal muscle that preserves most of the ECM molecules, including the proteoglycans. This decellularised skeletal muscle matrix was solubilized and examined for its ability to support C2C12 myoblast proliferation and differentiation under serum free culture conditions. The serum-free medium used was one that we developed specifically for C2C12 cell growth and differentiation on fibronectin substrates, and enabled us to assess the affect of the ECM on myoblast proliferation and differentiation in the absence of the confounding variables present in serum. The present study indicated that the complex muscle matrix supported myoblast differentiation, with differentiation occurring earlier on this matrix than on collagen I to result in the formation of well aligned myotubes. Under serum free conditions the myoblasts secreted and organised their own ECM when placed on a substrate and this endogenous matrix will also contribute to the myoblast’s local microenvironment. On 3D muscle matrices, in serum-free conditions, C2C12 cells aligned and orientated in patterns corresponding to the structure of the underlying muscle matrix. This suggests that although “generalised” ECM scaffolds can assist in muscle repair, the correct muscle ECM may facilitate better cell organisation and differentiation.

## Materials and Methods

### Muscle sampling & ethics

Female C57Bl/6J mice aged 3 months were obtained from the Animal Resource Centre (Perth, Australia) and were acclimated for one week following delivery. All procedures were carried out in strict accordance with the guidelines of the National Health and Medical Research Council of Australia and were approved by the Curtin Animal Ethics Committee (AEC 2011-73A). Mice were anesthetized with 2% isoflurane and euthanized by cervical dislocation. Rats euthanized by intraperitoneal overdose (50 mg/100 g body weight) of pentobarbitone sodium (Lethabarb) were obtained from Dr Christine Cooper, Curtin University. Following euthanasia, quadriceps muscles from both legs of the mice and rats were removed. Muscles were either snap frozen in liquid nitrogen, or embedded for cryosectioning and frozen using isopentane cooled with liquid nitrogen. All samples were stored at -80°C.

### Decellularization of skeletal muscle sections

10 μm thick frozen sections were cut using a cryostat, mounted on silanated glass slides (ProScitech, Thuringowa, Australia) and decellularized using either trypsin, SDS or phospholipase A2 (PLA_2_) (all from Sigma Aldrich, St Louis, MO). For the trypsin method [18], muscle sections were rinsed in PBS for 2 h and incubated in 0.05% trypsin for 20 min before the trypsin was inhibited by Dulbecco’s modified Eagle’s medium (DMEM)(Life Technologies, Carlsbad, CA) containing 10% fetal bovine serum (FBS) (HyClone, Thermo Scientific, Waltham, MA) for 1 h. Sections were then washed in PBS/1% Triton X-100 for 30 min and PBS for 1 h. SDS-based decellularisation was performed by rinsing muscle sections in distilled water for 2 h before treatment with 0.2% SDS in PBS/50 mM EDTA and 1 x cOmplete protease inhibitor (Roche, Mannheim, Germany) for 75 min at 4°C with buffer being replaced every 20 min. Sections were then washed with 2 M NaCl/50 mM EDTA/1x protease inhibitors/20 mM Tris, pH 7.4 for 10 min at 4°C followed by 100 mM NaCl/20 mM Tris for 1 h at 4°C. Finally they were washed twice, for 30 min, with 20 mM Tris/0.15M NaCl buffer. For the PLA_2_ method [24] muscle sections were rinsed in PBS for 30 min and incubated in a phospholipase A_2_ (PLA_2_ 170 U/ml), 0.5% sodium deoxycholate solution in 20 mM Tris buffer (pH 8.0)/0.15M NaCl containing 1x cOmplete protease inhibitor for 30 min. Sections were washed in 3.4 M NaCl/20mM Tris for 2 h, washed twice in PBS for 5 min, treated with DNaseI (75 U/ml) (Promega, Madison, WI) in 1x DNase I reaction buffer (40 mM Tris-HCl pH 8.0, 10 mM MgSO_4_ and 1.0 mM CaCl_2_) for 1 h at 37°C and then washed 3x in PBS for 15 min each. Unless otherwise stated, all chemicals and solutions used in these processes were purchased from Sigma Aldrich.

DNA in muscle sections was visualized using Vectashield mounting medium containing 4’,6-diamidino-2-phenylindole (DAPI) (Vector Laboratories, Burlingame, CA) and images were captured using a Zeiss Axioskop fluorescent microscope (Carl Zeiss, Oberkochen, Germany) and Spot Advanced software (SPOT Imaging solutions, Sterling Heights, MI).

### Immunohistochemistry—muscle sections

Frozen muscle sections (10 μm) were thawed and fixed in 4% paraformaldehyde/PBS for 10 min and blocked in 10% FBS/1% bovine serum albumin (BSA) in PBS (blocking solution) overnight at 4°C. Sections were washed 3 times with PBS and incubated for 2 h in primary antibody (Table 1) diluted in blocking solution, before being washed with PBS and incubated in secondary antibody (Table 2) diluted in blocking solution. Sections were then washed and mounted in Vectashield. Images were captured with a Zeiss Axioskop fluorescent microscope using Spot Advanced software. Images were acquired at the same gain and exposure settings and were processed using the same contrast and brightness settings. The blocking solution for anti-decorin antibody was 10% normal donkey serum in PBS. The isotype control antibodies were rat IgG2a, mouse IgG, rabbit IgG (all from, Life Technologies), and sheep IgG (Jackson ImmunoResearch).

**Table 1:** Primary antibodies.

Antibody	Species, Isotype	Clonality and Source Manufacturer.
collagen type I	Rabbit IgG	PolyclonalAbcam
collagen type III	Rabbit IgG	PolyclonalAbcam
collagen type IV	Rabbit IgG	PolyclonalAbcam
collagen type VI	Rabbit IgG	PolyclonalAbcam
Fibronectin	Rabbit IgG	Polyclonal, Abcam
Fibronectin	Mouse IgG1	Monoclonal, EP5, Santa Cruz
Laminin α2	Rat IgG_1_	Monoclonal, 4H8-2Abcam
Myosin heavy chain (slow)	Mouse IgG	Monoclonal, NOQ7.4.D, Millipore
Decorin	Sheep IgG	PolyclonalAbcam
Perlecan	Rabbit IgG	CCN-1, Polyclonal, John Whitelock [47]
EV3C3	Mouse	Phage display, Toin Van Kuppevelt [48, 49]
beta-tubulin	Rabbit IgG	Polyclonal, Abcam

**Table 2:** Secondary antibodies.

Name, Immunoglobulin	Conjugated	Manufacturer
Goat anti-mouse	HRP	Dako
Goat anti-rabbit	HRP	Dako
Rabbit anti-rat	HRP	Dako
Goat anti-rabbit	FITC	Sigma
Goat anti-rabbit	Alexa-fluor 488	Molecular Probes
Goat anti-rabbit	Alexa-fluor 546	Molecular Probes
Goat anti-rat	FITC	Sigma
Goat anti-mouse	Alexa-fluor 488	Molecular Probes
Donkey anti-sheep	Dylight 549	Jackson Immunoresearch
Rabbit, anti-VSV		Sigma Aldrich

### Whole muscle decellularization and solubilisation of skeletal muscle ECM

Rat tissue was used for whole muscle decellularization to obtain sufficient material for myoblast culture studies. Muscle was sliced into 0.5 mm pieces and rinsed in PBS containing 1x cOmplete protease inhibitor (Roche) for 2 h at 4°C. Muscle pieces were then treated with PLA_2_ (170 U/ml) 0.5% sodium deoxycholate in 20 mM Tris buffer (pH 8.0)/0.15M NaCl containing the protease inhibitor for 18 h at room temperature (RT) until transparent. Tissue samples were washed with 3.4 M NaCl/20 mM Tris/1x protease inhibitor for 24 h at 4°C (buffer changed every 8 h) and then rinsed in PBS for 1 h at 4°C. The samples were treated with DNAse I (75 U/ml) in DNase I reaction buffer at 37°C for 24 h, washed in PBS for 12 h at 4°C (3 buffer changes) and stored at 4°C until used. Decellularized samples for solubilisation were air-dried then vacuum dried for 3 h (Integrated SpeedVac, Thermo Scientific) and solubilized in 0.1 M acetic acid/20 mM EDTA for 7 days at 4°C. The protein concentration was measured using the Pierce BCA protein assay kit (Pierce Biotechnology, Rockford, IL).

### DNA quantification in whole muscles

Forty 10 μm sections of native and decellularised muscle were collected in 1.5 ml microtubes in 300 μl of PBS. DNA was extracted using the Masterpure kit (Epicentre, Madison, WI) according to the manufacturer’s instructions. Briefly, the muscle was incubated in 600 μl of lysis solution containing 0.1 mg proteinase K (Sigma-Aldrich) at 65°C for 45 min before being cooled to 37°C and incubated with 10 ng RNase A (Epicentre) for 30 min. Samples were kept on ice for 5 min, 300 μl of MPC protein precipitation reagent was added and the mixture centrifuged at 10,000 g for 10 min at 4°C. The supernatant was collected and DNA precipitated by adding 1 ml of isopropanol and centrifuging at 10,000g for 10 min at 4°C. The pellet was rinsed in 70% ethanol, dried and resuspended in 35 μl of TE (10 mM Tris (pH 8.0), 1 mM EDTA) buffer. DNA was quantified using a Nanodrop Spectrophotometer (Thermo Scientific, Waltham, MA) and electrophoresed on a 1.5% agarose gel.

### SDS-PAGE and Western blotting

Solubilised muscle extracts were separated by SDS-PAGE on a 7.5% gel, using 10 μg of collagen I as a standard. Gels were stained with Coomassie Brilliant Blue-R 250 (Bio-Rad, Hercules, CA), and destained in 40% methanol/10% glacial acetic acid/50% ddH_2_O. Gels were imaged using a ChemiDoc MP System (BioRad).

For Western blots, solubilised muscle extracts (40 μg) were separated by SDS-PAGE on a 4–15% Mini-PROTEAN TGX gradient gel (Bio-Rad) and proteins transferred to Immobilon-P PVDF membrane (Millipore, Billerica, MA). The membrane was blocked in 5% skimmed milk/0.1% Tween-20/PBS overnight at 4°C and incubated in primary antibodies. Membranes were washed in 0.1% Tween-20 in PBS (PBST) and incubated in PBST containing anti-rabbit, anti-mouse or anti-rat HRP-conjugated secondary antibodies (dilution, 1:2000 for anti-rabbit and 1:1000 for anti-mouse and rat). Membranes were washed, incubated in Western Lightning Plus-ECL substrate (Perkin Elmer, Waltham, MA) for 5 min, then imaged using ChemiDoc MP System.

### Scanning electron microscopy

Muscle sections from mice and rats were fixed in 4% paraformaldehyde for 30 min at RT and dehydrated in ethanol solutions of increasing concentration (30%- 100%), for 15 min per concentration. Muscle sections were dried at RT, mounted on aluminium stubs and sputter coated with platinum (5 nm) using a 208HR sputter coater (Cressington, Redding, CA). Data were collected using an EVO Scanning Electron Microscope (Carl Zeiss).

### Cell culture

The murine myoblast cell line, C2C12 (ATCC, Manassas, VA) was subcultured in DMEM, Life Technologies, Carlsbad, CA) supplemented with 10% FBS (HyClone, Thermo Scientific), 10 mM Hepes, 1 mM sodium pyruvate and 2 mM glutamine (all from Life Technologies). C2C12 myoblasts were passaged at 60–70% confluency and cells less than 20 passages were used in all experiments.

### Serum-free Proliferation and Differentiation assays

Wells of a 24 well tissue culture plate (Nunc, Thermo Fisher Scientific) were coated with 10 μg/cm^2^ of solubilised ECM from acellular muscle, 10 μg/cm^2^ of collagen I or 5 μg/cm^2^ of fibronectin (both from Sigma-Aldrich), incubated at 37°C for 2 h, washed with PBS and sterilized under UV light. C2C12 myoblasts (7x10^3^ cells/well) were plated in serum free proliferation medium comprising DMEM/Ham’s F10 medium (Sigma-Aldrich) in a 1:1 ratio, 50 ng/ml epidermal growth factor (EGF), 25 ng/ml FGF-2, 1.0 μM/ml dexamethasone, 0.12 IU/ml insulin, 1.0 μg/ml heparin and 0.5% BSA. Growth factors were from Peprotech (Rocky Hill, NJ), dexamethasone, insulin and BSA were from Sigma-Aldrich and heparin was from Celsus Laboratories (Cincinnati, OH). Cells were maintained in serum free proliferation medium for 4 days and then transferred to differentiation medium (DMEM/F10 (1:1) containing 50 ng/ml EGF, 25 ng/ml insulin growth factor (IGF-1, Peprotech), 1.0 μM/ml dexamethasone and 0.5% BSA). Controls were cells grown in proliferation medium comprising DMEM/F10/10% FBS and differentiation medium comprising DMEM/F10/2% horse serum. For proliferation assays, cells were harvested using 0.05% trypsin/1mM EDTA and counted using a Z Series COULTER COUNTER (Beckmann Coulter, Indianapolis, IN). Cell count data is representative of four independent experiments.

For immunofluorescence, C2C12 cells were cultured on glass coverslips coated with collagen I (10 μg/cm^2^), fibronectin (5 μg/cm^2^) or solubilized muscle matrix (10 μg/cm^2^). On days 4, 6 and 8 of differentiation cells were fixed in 4% paraformaldehyde/PBS, permeabilized in 0.1% Triton X-100/PBS and blocked in 10% FBS/1% BSA/PBS (blocking solution) for 1 h at RT. Cells were washed with PBS and incubated for 2 h in anti-slow muscle myosin heavy chain (MyHCB) antibody, washed with PBS and incubated in Alexa Flour 488 conjugated goat anti-mouse antibody for 1 h and washed. Antibodies were diluted in blocking solution. The samples were mounted in a DAPI-containing mounting medium. Images were captured with Zeiss Axioskop fluorescent microscope using Spot Advanced software. Myotube width was measured using Image J [25]. Fifty myotubes per treatment were measured from randomly selected MyHC positive myotubes from 6 non-overlapping fields of view. Three measurements were made along each myotube and the mean width calculated. The number of nuclei per myofibre (50 myofibres per treatment) was assessed by manual counting.

### Etching of coverslips

Round glass coverslips (13 mm^2^) (Knittel Glass, Braunschweig, Germany) were stored in 100% ethanol before etching. Coverslips were treated with etch solution (2.5M NaOH in 60% ethanol) for 30 min at RT. After treatment, the coverslips were washed extensively with ddH_2_O, dried and sterilized under UV light.

### Quantitative real time PCR (qPCR) of muscle genes

RNA was isolated from C2C12 myoblasts using the ISOLATE RNA KIT (Bioline, Alexandria, Australia) as per the manufacturer’s instructions. RNA concentration and purity was assessed using a Nanodrop spectrophotometer and the LabChip GX II (Perkin Elmer, Waltham, MA). All samples had an RNA quality score (RQS) of > 8.0. Reverse transcription was performed on 300 ng of RNA using the Tetro cDNA synthesis kit (Bioline). qPCR reactions were performed using SensiFAST SYBR Lo-Rox kit (Bioline), with triplicate reactions containing 5 μl SYBR Green Lo-RoX Mix, 2 μl template cDNA, 1 μl forward/reverse primer (25 ng/μl) and 2 μl RNase free H_2_O. Reactions were performed on a ViiA7 Real-Time PCR system (Applied Biosystems, Life Technology) with a fast 96-well block using the cycling conditions: denaturation at 95°C for 2 min, 40 cycles of denaturation at 95°C for 5 sec and annealing/extension at 60°C for 20 sec, followed by a melt step ranging from 55–95°C. Primers were selected for four reference genes and seven genes of interest (Table 3) using the PrimerBank database [26] and purchased from Geneworks (Hindmarsh, Australia). Of the reference genes, *ACTAB* and *SDHA* were found to have the most stable expression by Normfinder [27] and Bestkeeper [28] software. Expression levels for *Myf5*, *MYOG*, *MYH1*, *MYH3*, *MYH7*, *ACTA1* and *ACTC1* were normalized to *SDHA* expression values and fold change determined using 2^-delta delta Ct^ method with baseline expression at day 1. Mean ± SE of 4 replicates is shown. Statistical significance was determined using a two-tailed t-test at p<0.05.

**Table 3:** Primer and gene information for qPCR.

Gene Name^a^		Sequence (5’-3’)	NCBI reference number	PrimerBank ID number	BP^b^
SDHA	FR	TGG GGA GTG CCG TGG TGT CA TGC CCC GTA GCC CCC AGT AG	NM_023281		100
ACTAB	FR	GTG ACG TTG ACA TCC GTA AAG A GCC GGA CTC ATC GTA CTC C	NM_007393	145966868c1	245
TBP	FR	CCT TGT ACC CTT CAC CAA TGA C ACA GCC AAG ATT CAC GGT AGA	NM_013684	172073170c2	119
GAPDH	FR	AGG TCG GTG TGA ACG GAT TTG TGT AGA CCA TGT AGT TGA GGT C	NM_008084		139
MYOG	FR	CGA TCT CCG CTA CAG AGG C GTT GGG ACC GAA CTC CAG T	NM_031189	162287254c3	137
MYF5	FR	GCC TTC GGA GCA CAC AAA G TGA CCT TCT TCA GGC GTC TAC	NM_008656	240120094c2	187
ACTA1	FR	CCC AAA GCT AAC CGG GAG AAG GAC AGC ACC GCC TGG ATA G	NM_009606	133893192c1	89
ACTC1	FR	CTG GAT TCT GGC GAT GGT GTA CGG ACA ATT TCA CGT TCA GCA	NM_009608	14192922a1	173
MYH1	FR	CGG AGT CAG GTG AAT ACT CAC G GAG CAT GAG CTA AGG CAC TCT	NM_030679	82524273c2	153
MYH3	FR	ATG AGT AGC GAC ACC GAG ATG ACA AAG CAG TAG GTT TTG GCA T	NM_001099635	153792648c1	119
MYH7	FR	ACT GTC AAC ACT AAG AGG GTC A TTG GAT GAT TTG ATC TTC CAG GG	NM_080728	18859641a1	114

^a^SDHA- Succinate dehydrogenase complex, subunit A; ACTAB- beta-actin; TBP- Tata binding protein; GAPDH- Glyceraldehyde-3 phosphate dehydrogenase; MYF5- Myogenic factor 5; MYOG-Myogenin; ACTA1- Actin, alpha 1, skeletal muscle; ACTC1- Actin, alpha 1, cardiac muscle; MYH1- Myosin heavy chain-1; MYH3- Myosin heavy chain-3; MYH7- Myosin heavy chain-7.

^b^BP—length in base pairs.

### Three-dimensional cell culture

Longitudinal and transverse cut samples of acellular rat muscle were placed in wells of a 24 well plate. C2C12 cells were harvested and 1x10^4^ cells re-suspended in 50 μl serum free medium were added to scaffolds and allowed to adhere for 30 min at 37°C. Scaffolds were transferred to new wells, 1.5 ml of media was added and cells were cultured for 7 and 14 days, with medium replaced every third day. Cells and scaffolds were stained with Kwik Diff stain (Thermo Fisher Scientific) which stains cell nuclei red and cell membranes and cytoplasm blue.

Click-iT EdU (Life Technologies) cell proliferation assay was performed at day 3 of proliferation and day 6 of differentiation. Half the medium was replaced by 750 μl of medium containing 20 μM EdU and incubated for 24 h. Medium was removed, the scaffolds fixed in 4% paraformaldehyde/PBS, washed and treated with 0.5% TX-100/PBS for 10 min. Scaffolds were washed and incubated in 100 μl of Click-iT reaction cocktail for 30 min at RT, then washed again, incubated in DAPI/PBS (1 μg/ml) for 10 min and imaged using a Nikon A1+ confocal microscope (Nikon, Tokyo, Japan) using NIS-Elements AR analysis version 4.10 software. Z-stack images (69 at 2 μm steps) were taken and merged to generate a single image.

## Results

### Development of the decellularisation procedure

Following decellularisation of quadriceps muscle by three methods (trypsin digestion, SDS treatment or phospholipase A2 (PLA_2_) treatment), DAPI staining was used to visualize DNA, to indicate the extent of cell removal. No DAPI staining was seen following trypsin or PLA_2_ treatment but staining was present after 0.2% SDS treatment (S1A Fig). In addition, DNA extracted from untreated and decellularised muscle (of equal weights before treatment) was separated on an agarose gel and the intensity of the bands quantified. A band of genomic DNA was visible in the control lanes and muscle decellularised by SDS treatment, but there was no DNA in the lanes containing extracts from muscle decellularized by trypsin or PLA_2_ (S1B Fig). Spectrophotometric analysis revealed a 58%, 36% and 82% loss of DNA by the trypsin, SDS and PLA_2_ methods, respectively.

**Fig 1:**
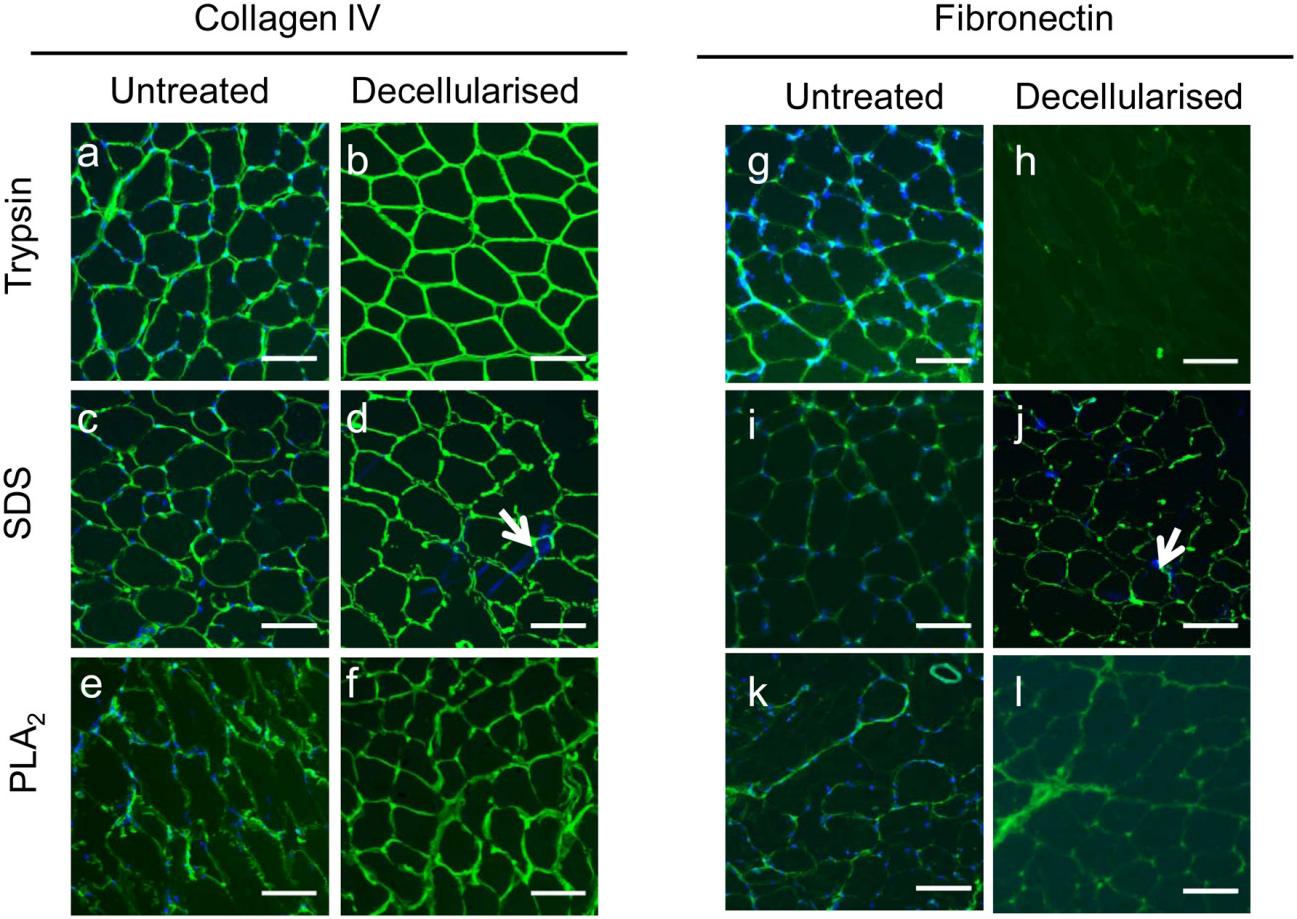
Differential preservation of collagen IV and fibronectin in sections treated with trypsin, SDS and PLA_2_. Quadriceps muscle sections treated with trypsin (b, h), SDS (d, j) and PLA_**2**_ (f, l), and untreated controls (a, c, e, g, i, k), were fixed with 4% paraformaldehyde and stained with rabbit polyclonal antibodies against collagen IV and fibronectin followed by a goat anti-rabbit FITC conjugated secondary antibody. Nuclei are stained with DAPI (blue). Arrows indicate nuclear material remaining after decellularisation in SDS treated samples. Scale bar—100 μm.

Western blots for the cellular proteins myosin heavy chain (MyHCB) and β-tubulin revealed two distinct bands at 220 kDa and 160 kDa for myosin and one band at 50 kDa for β-tubulin in the control sample, but no bands in the decellularised sample (S1C Fig). Immunostaining of muscle sections for collagen IV and fibronectin was also used to evaluate ECM preservation. Comparable collagen IV immunostaining to that of the controls was obtained following decellularisation regardless of the method (Fig 1a–1f). In contrast, fibronectin epitopes were preserved in tissue treated with SDS or PLA_2_ (Fig 1, compare i and j; k and l) but were virtually absent in trypsin treated sections (Fig 1, compare g and h). Collectively, these data indicate that PLA_2_ treatment was the most effective of the decellularization methods tested and this procedure was used for all subsequent experiments.

### Characterization of matrix after decellularisation

**Fig 2:**
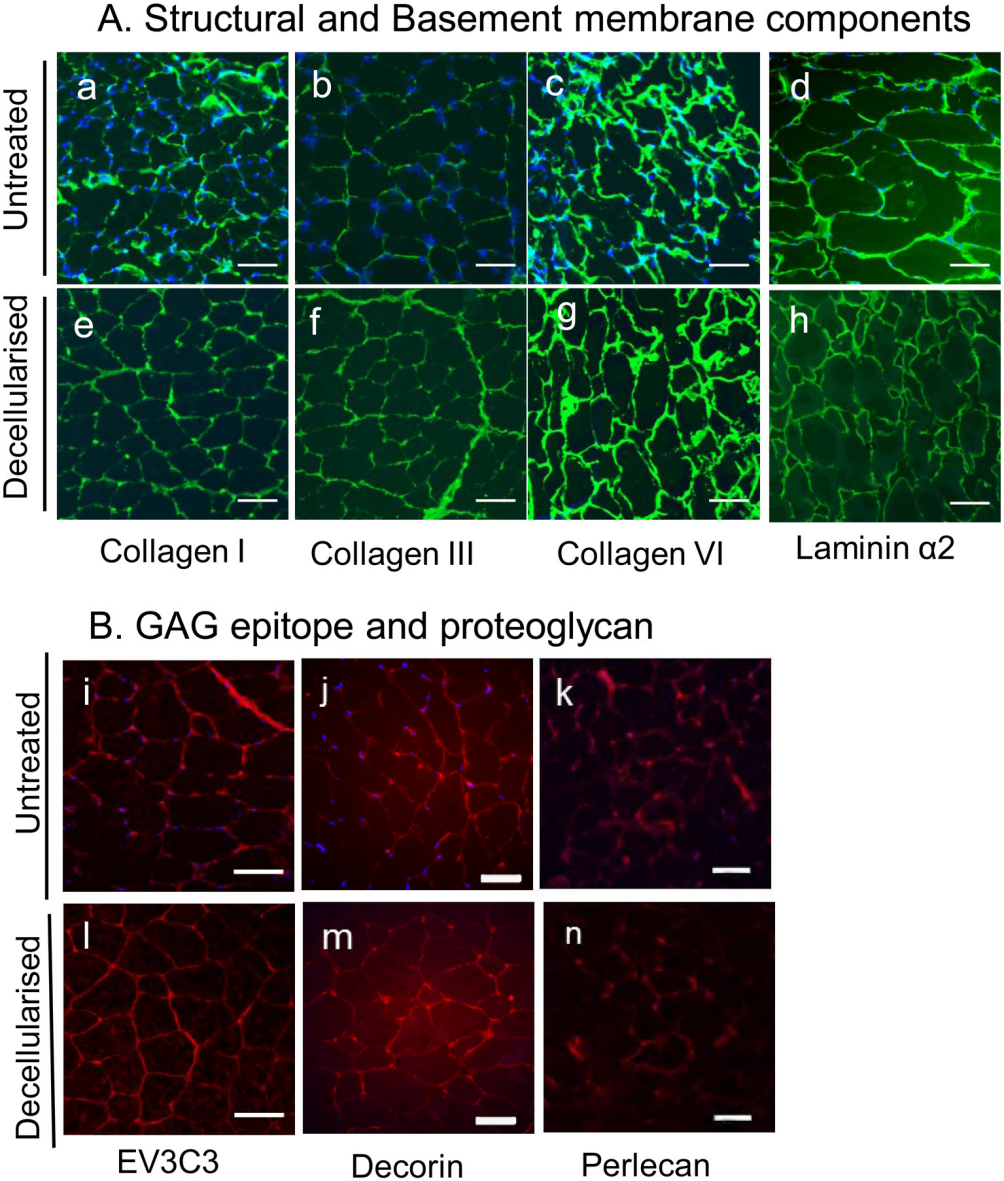
ECM components are preserved in PLA_2_ decellularised muscle sections. Untreated (a–d, i–k) and PLA_**2**_ decellularised (e–h, l–n) sections were immunostained. The antibodies against collagen I (a, e), collagen III (b, f), collagen VI (c, g) and perlecan (k, n) were rabbit polyclonals and the second antibody was goat anti-rabbit FITC. Antibodies against laminin α2 (d, h) and decorin (j, m) were rat and sheep polyclonals respectively, and secondary antibodies were goat anti-rat and donkey anti-sheep FITC. The mAb, EV3C3, (i, l) recognises a heparan sulfate epitope [46] and contains a vesicular stomatitis virus Glycoprotein (VSV-G) tag. A rabbbit anti-VSV antibody followed by goat-anti-rabbit FITC was used. Nuclei are stained with DAPI (blue). Scale bars—100 μm for a-h, and 50 μm for i-n.

Immunostaining indicated collagens I and III were well preserved in the endomysium after PLA_2_ decellularisation of muscle sections, with an increase in the fluorescence intensity of staining following decellularisation, compared with controls (Fig 2a, 2b, 2e and 2f). Strong staining similar to that of intact muscle was also observed for the basement membrane components collagen VI (Fig 2c and 2g) and laminin α2 (Fig 2d and 2h) in the decellularised muscle sections. Following decellularization, the heparan sulphate epitope recognised by the antibody EV3C3 was detected (Fig 2I and 2l) and immunostaining of the proteoglycans perlecan (Fig 2j and 2m) and decorin (Fig 2k and 2n) indicated preservation of these molecules.

**Fig 3:**
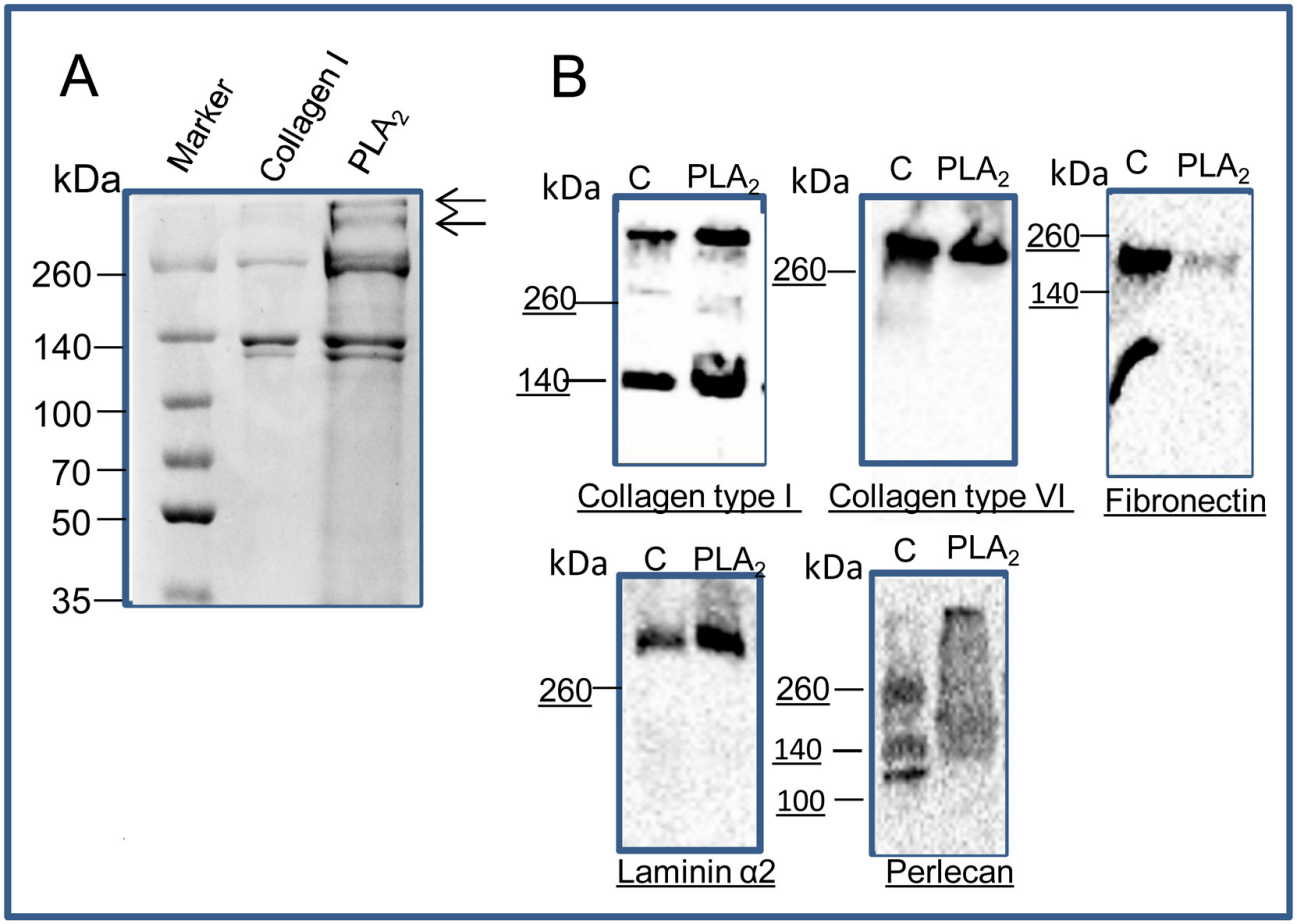
SDS-PAGE and Western blotting indicates preservation of matrix proteins. (A) After PLA_**2**_ decellularisation ECM proteins were solubilised, resolved on a 7.5% SDS-PAGE and visualized by Coomassie Blue staining: PLA_**2**_ decellularised matrix (lane 3) and collagen 1 (lane 2). (B) Muscle extracts (untreated and PLA_**2**_ treated) were resolved on 4–15% gradient gels and membranes were probed with antibodies against collagen I, collagen VI, fibronectin, and perlecan (all rabbit polyclonals) and laminin α2 (rat, clone 4H8-2). Secondary antibodies were anti-rabbit or anti-rat conjugated to HRP, and blots were visualized using ECL. C: untreated muscle extract, PLA_**2**_: decellularise muscle extract.

Solubilized ECM extracted from PLA_2_ decellularised muscle was resolved by SDS-PAGE. Coomassie Blue staining revealed the ECM extract contained high molecular weight proteins and proteins of comparable size to collagen I (Fig 3a). The lack of low molecular weight proteins indicated a loss of cytoplasmic proteins following decellularization. These extracts were analysed using Western blotting to determine if collagen I, collagen VI, laminin α2, perlecan and fibronectin were preserved (Fig 3b). Two bands were evident on the collagen I blot, one at ~140 kDa, probably the α1 chain, and a possible trimer above 260 kDa. The collagen VI blot had a band above 260 kDa that was similar in intensity for extracts from untreated and decellularised muscle. Fibronectin was detected as a 200 kDa band, although with a lower signal in the decellularized extract compared to the untreated extract. A high molecular weight band (~400kDa) bound the Laminin α2 antibody in lanes of both decellularised and untreated muscle extracts. Multiple bands were revealed in perlecan blots of the untreated extract, but a single high molecular weight band was present in the decellularised extract.

**Fig 4:**
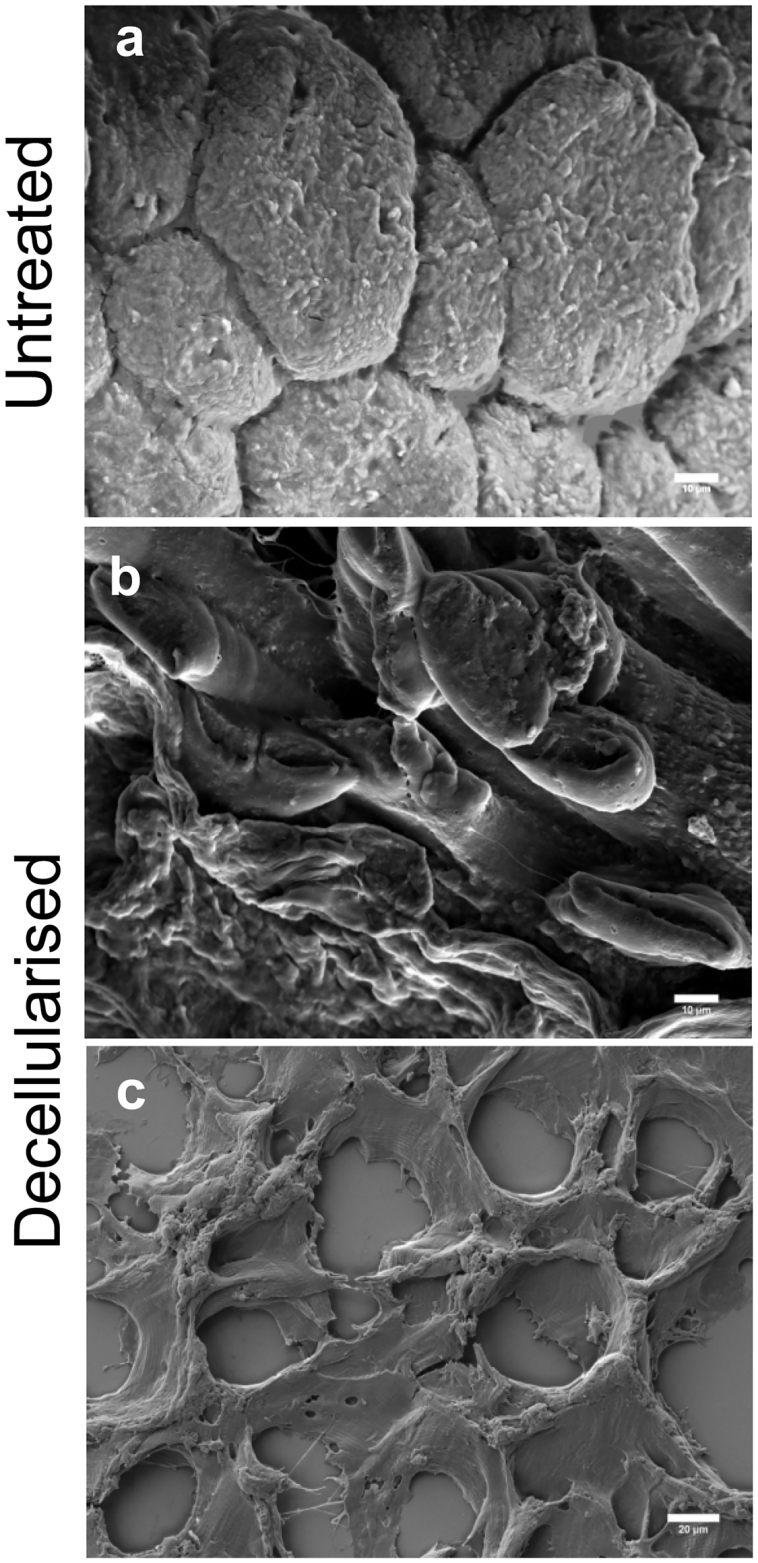
Structural integrity of decellularized muscle tissue. Scanning electron micrographs of 0.5 mm thick rat muscle sections (a, b) and 60 μm thick murine muscle sections (c). Images are representative of five samples. Scale bars are 10 μm (a–b) and 20 μm (c).

Scanning electron microscopy confirmed the overall structural integrity of the decellularised matrices (Fig 4). Control muscle showed a hexagonal, compact fibre arrangement (Fig 4a) while hollow cylindrical structures were evident in the decellularized samples, revealing the tubular nature of the endomysial ECM (Fig 4b and 4c). Collectively, these data supported the conclusion that the decellularised matrix prepared using PLA_2_ retained its integrity.

### Proliferation and differentiation of C2C12 myoblasts on different matrices

**Fig 5:**
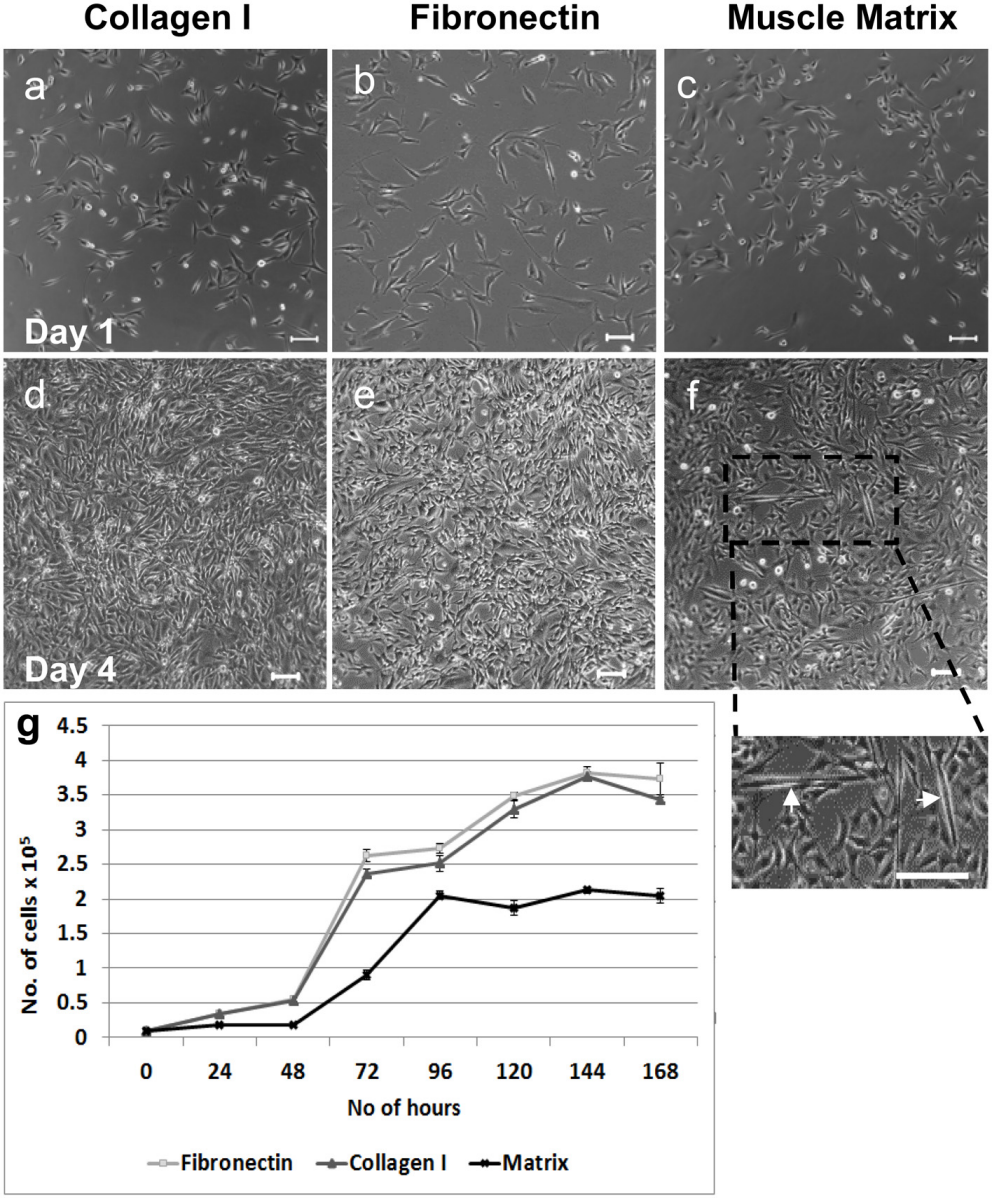
ECM proteins support C2C12 myoblast proliferation in serum free cultures. (A) Phase contrast images show C2C12 myoblasts cultured on collagen I (a, d), fibronectin (b, e) and solubilized muscle matrix (c, f) after 1 and 4 days in serum free culture. Scale bars are 150 μm. A section of “f” is shown at a higher magnification (Scale bar = 200 μm). Proliferation of C2C12 cells on protein substrates in serum free medium was assessed by a direct cell count (g). Data are mean ± SD of 4 independent experiments.

The murine myoblasts C2C12s were cultured on tissue culture plastic coated with collagen 1, fibronectin or solubilized rat ECM in serum free medium for 7 days (Fig 5). C2C12 myoblasts are commonly used to investigate myoblast fusion and myotube formation. Rat quadriceps matrix was used for these experiments, as it was not possible to obtain sufficient matrix material from the smaller mouse quadriceps.

In the absence of a matrix substrate, C2C12 cells clustered and adhered poorly to the tissue culture plastic and their proliferation and differentiation was impaired (data not shown). In contrast, C2C12 myoblasts adhered to, spread and proliferated on all matrices (Fig 5a–5f). They proliferated at a similar rate on collagen I and fibronectin, but grew more slowly on muscle matrix, with cell numbers plateauing at day 4 (Fig 5g), when they began to differentiate. Small myotubes can be readily seen in cultures on muscle matrix at day 4 (Fig 5f), but myotubes were not present in cultures on collagen I or fibronectin at this stage.

**Fig 6:**
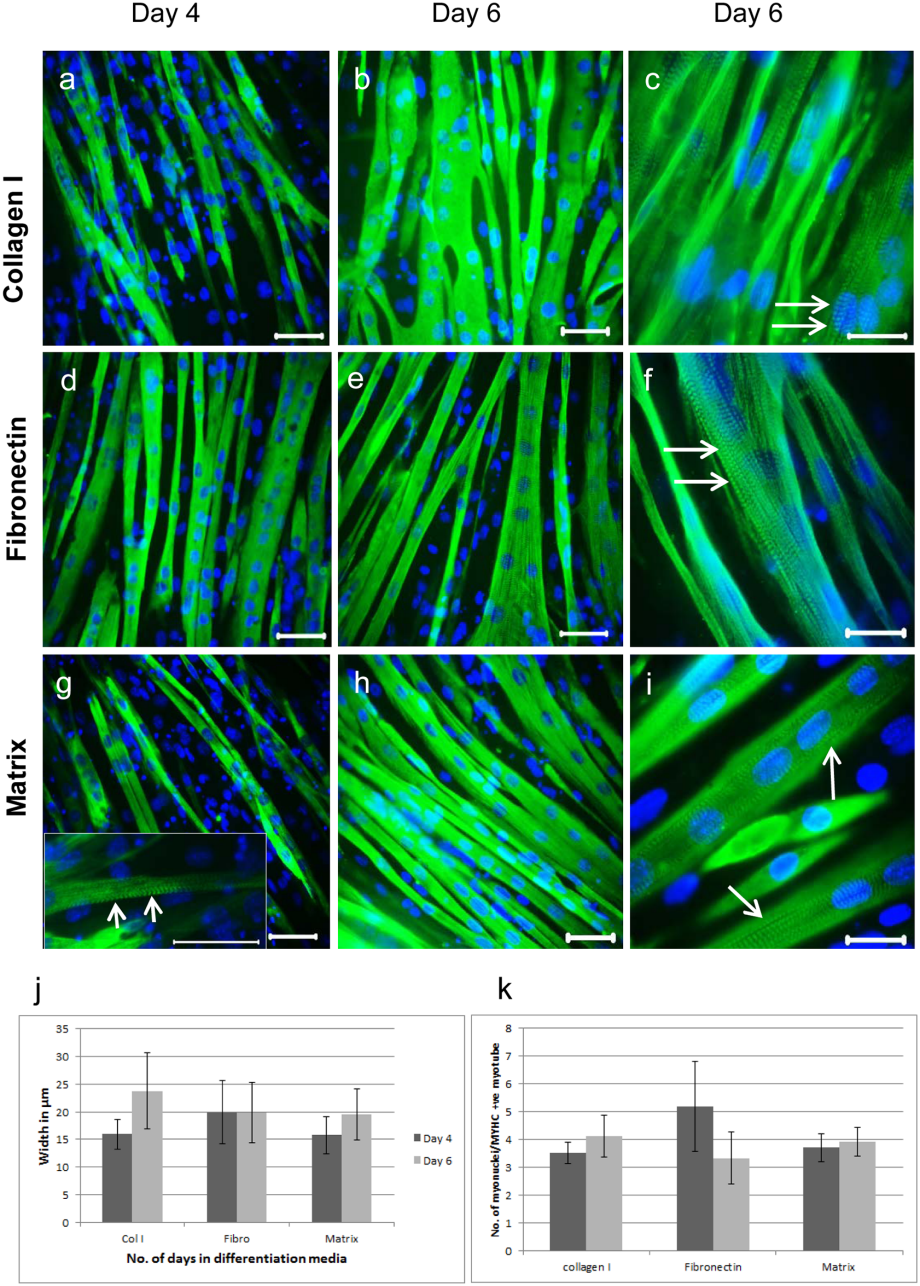
Immunofluorescent staining of mature C2C12 myotubes. C2C12 myoblasts were cultured for 4 and 6 days in differentiation medium on collagen I (a–c), fibronectin (d–f) and muscle matrix (g–i), and visualized using the mouse anti-MyHCB mAb (clone NOQ7.5.4D) and goat anti-mouse AF488. Arrows indicate striations (c, f, g and i). Nuclei are stained with DAPI (blue). Scale bars are 50 μm for a–b, d–e, g–h and g insert; and 25 μm for c, f and i. Myotube width was measured using ImageJ software (j), values are the mean and SD of 50 myotubes.

Next, the differentiation of C2C12 myoblasts cultured on the various matrix substrates was examined. Cells were cultured in serum free proliferation medium until 90% confluent (day 4) and then switched into serum-free differentiation medium for a further 6–8 days. Immunostaining with a monoclonal antibody (mAb) to MyHCB (Fig 6a–6i) revealed the myoblasts had differentiated and fused to form myotubes on all substrates, although on collagen I the myotubes were less well aligned and more branched than on the other substrates. Myotube width increased from day 4 to day 6 in myoblasts cultured on collagen I and muscle matrices, while widths remained constant in myotubes formed on fibronectin over the same time period (Fig 6j). Overall, there was no difference in myotube width between the three substrates (Fig 6j). The number of nuclei per myofibre was also similar for all three substrates (Fig 6k) and by day 6 of differentiation, myotubes on all substrates developed striations. Striations were observed in myotubes on muscle matrix at day 4 of differentiation (Fig 6g), whereas this was not the case when C2C12 cells were grown and differentiated on collagen I or fibronectin.

### Gene expression of myogenic markers in C2C12 cells

**Fig 7:**
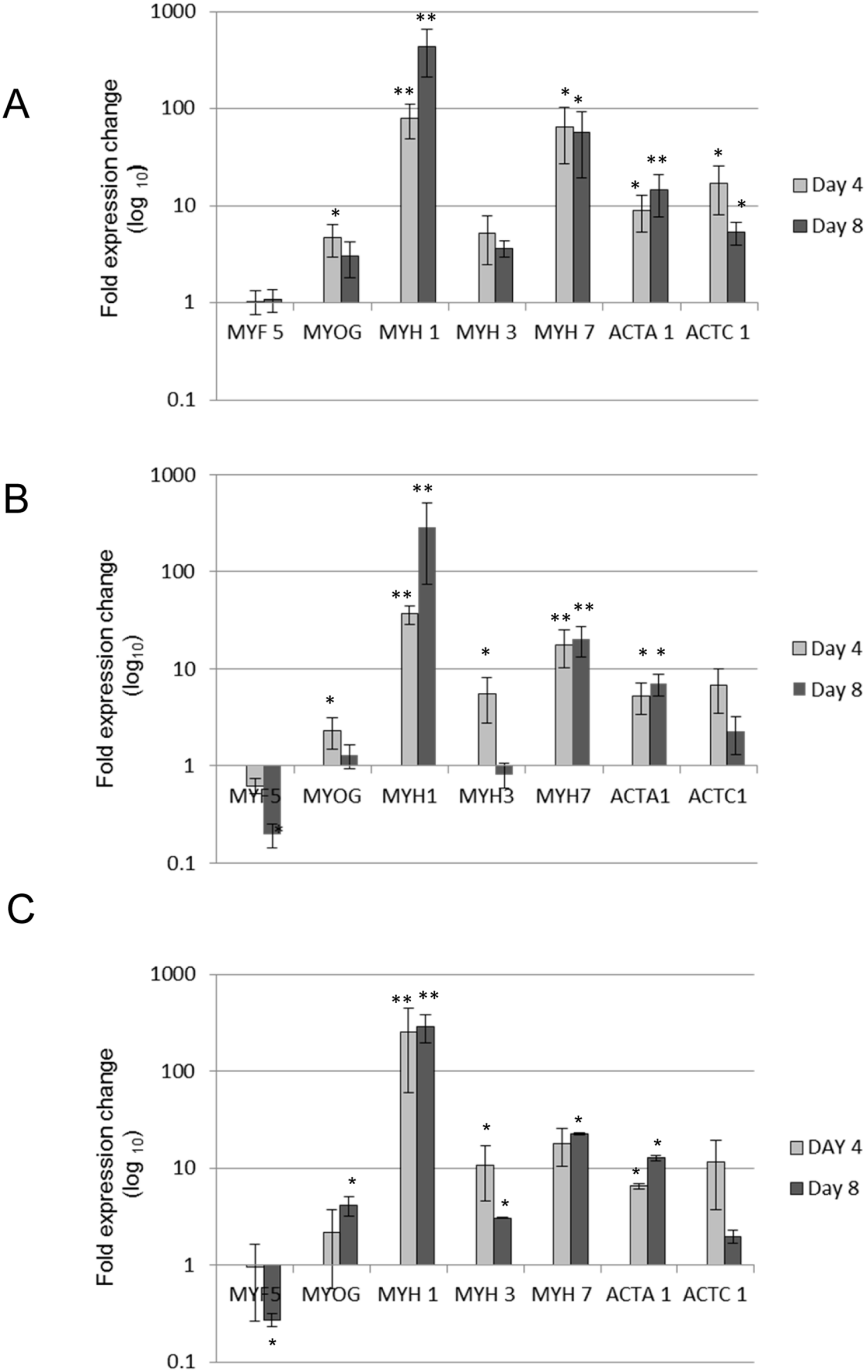
Quantitative Real time PCR analysis reveals muscle differentiation on matrix proteins. qPCR was used to assess the expression of markers (Table 3) in differentiating C2C12 cells grown in serum free medium on collagen I (a) fibronectin (b) and solubilised muscle matrix (c) at days 1, 4 and 8 of differentiation. Relative expression levels for *MYF5*, *MYOG*, *MYH1*, *MYH3*, *MYH7*, *ACTA1* and *ACTC1* were normalized to the Ct value of the reference gene (*SDHA*) and fold change was determined using the 2^-delta delta^ C_t_ method. Day 4 and Day 8 expression levels were normalized to Day 1 and log10 transformed. Mean ± SE of 4 biological replicates are shown. Significance was determined using a two-tailed t test at P<0.05*.

The expression of genes involved in different stages of myogenesis was assessed using qPCR. These genes were myogenic factor 5 (*Myf5*) and myogenin (*MYOG*), both transcription factors belonging to the myogenic regulatory factor (MRF) family; three isoforms of myosin heavy chain (*MYH1*, *MYH3* and *MYH7*); and two muscle actin isoforms (*ACTA1* and *ACTC1*). Of the four reference genes tested, SDHA showed the most stable expression across all conditions and was used as the reference gene for normalization of data. Gene expression by C2C12 cells on days 1, 4 and 8 of differentiation was assessed when C2C12 myoblasts were cultured on collagen I, fibronectin and muscle matrix (Fig 7).

*Myf5* showed similar levels of expression at days 1, 4 and 8 of differentiation on collagen I (Fig 7a). On fibronectin and muscle matrix surfaces, *Myf5* levels at day 8 were significantly lower than levels at day 1 (Fig 7b and 7c). *MYOG* mRNA increased on muscle matrix coated surfaces by approximately 4-fold between days 1 and 8 (Fig 7c). In contrast, on collagen I and fibronectin coated surfaces, *MYOG* levels peaked at day 4 before declining. An increase in the expression of *MYH1*, *MYH7* and *ACTA1* was observed from differentiation day 1 to day 4, and maintained at day 8 on all matrices. *MYH3* and *ACTC1*, which are expressed in immature muscle, increased from differentiation day 1 to day 4, before decreasing at day 8. A similar trend was seen on all matrices (Fig 7), although the decrease in *MYH3* expression was most pronounced in cells on fibronectin and muscle matrix. These data, plus the fact that on muscle matrix *MYH1* expression did not increase from day 4 to day 8, suggests that differentiation occurred earlier in cells cultured on muscle matrix compared to collagen I, but at about the same time as those on fibronectin.

### C2C12 cell adhesion on three dimensional (3D) muscle matrices

**Fig 8:**
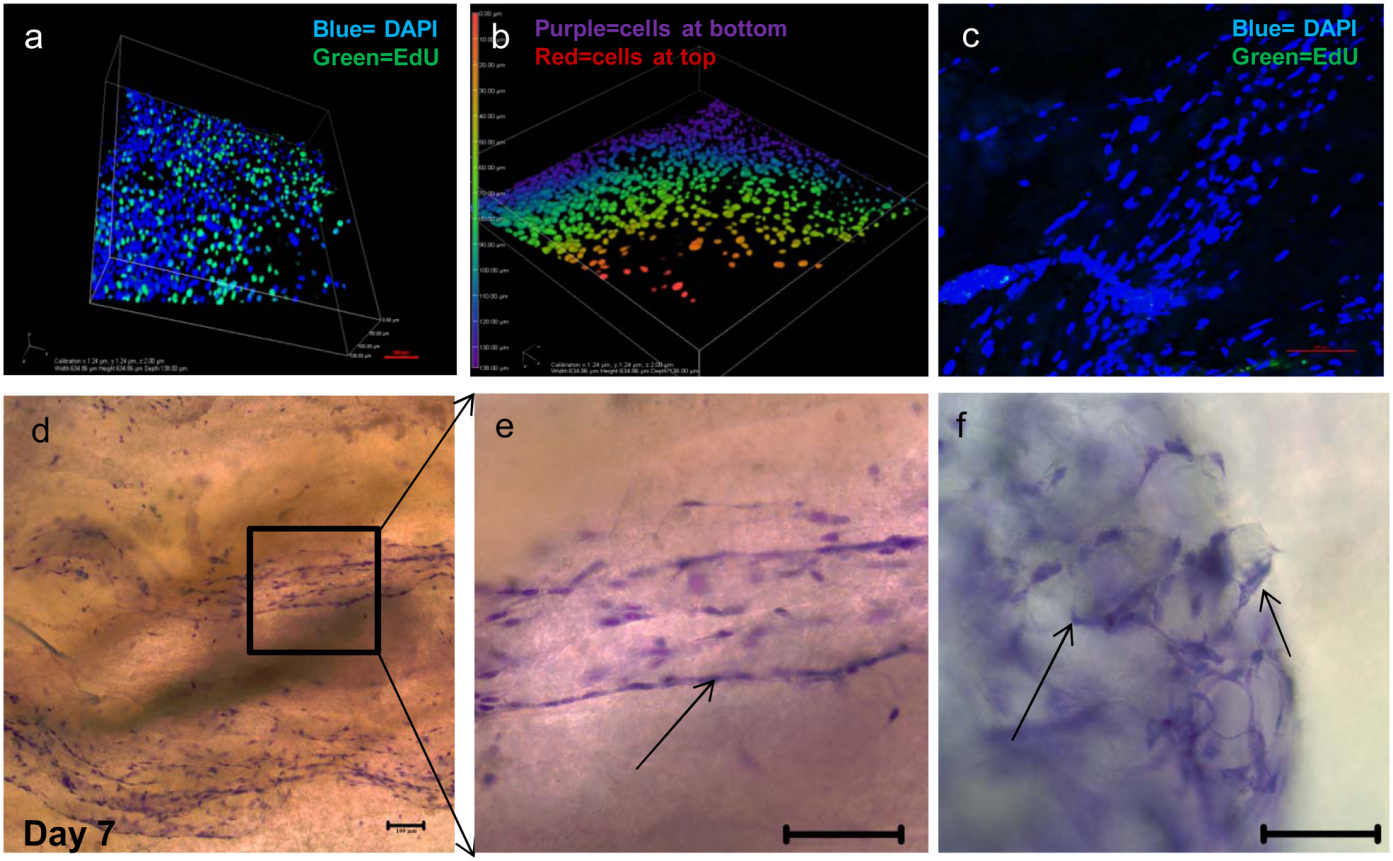
Decellularised 3D muscle scaffolds supported proliferation and migration of C2C12 cells. The click-iT EdU assay was performed on C2C12 myoblasts cultured for 3 days in proliferation media (proliferating cells: green, other cells: blue; scale bar 100 μm) (a), colour-coded image of the distribution of cells in the scaffold after 3 days (b). The EdU assay was performed on cells cultured for 3 days in proliferation media and 6 days in differentiation media (cells coloured as above; scale bar—100 μm)(c). Cells were grown for 7 days in proliferation media, stained with Kwik Diff (nuclei—red; cytoplasm and plasma membrane—blue) and imaged using bright field microscopy (Zeiss Axioskop), scale bar-100 μm (d–f). The arrows indicate C2C12 myoblasts aligning on longitudinal (e) or circular (f) tracks of ECM.

Decellularized 3D rat muscle was used to examine how an intact muscle matrix affected cell adhesion and proliferation. Cell proliferation in serum-free cultures on 3D matrices was assessed using a Click-iT EdU assay, which labels recently synthesized DNA with the coloured thymidine analogue EdU. Many cells were proliferating at day three after seeding (Fig 8a) and the 3D colour coded image of the muscle scaffold (Fig 8b) showed cells were distributed to a depth of 138 μm in the matrix. After 6 days of differentiation the EdU assay was repeated (Fig 8c). In this case the nuclei were elongated and there were no proliferating cells, suggesting that differentiation was occurring. Kwik Diff staining (Fig 8d and 8f) showed the myoblasts became organised on the 3D matrices and lined up either longitudinally (Fig 8d and 8e), or in circular patterns where the ECM had been cut in transverse section (Fig 8f). The cells appeared to be following the structure of the endomysial matrix.

### C2C12 cells differentiate and secrete matrix proteins on etched glass substrates

**Fig 9:**
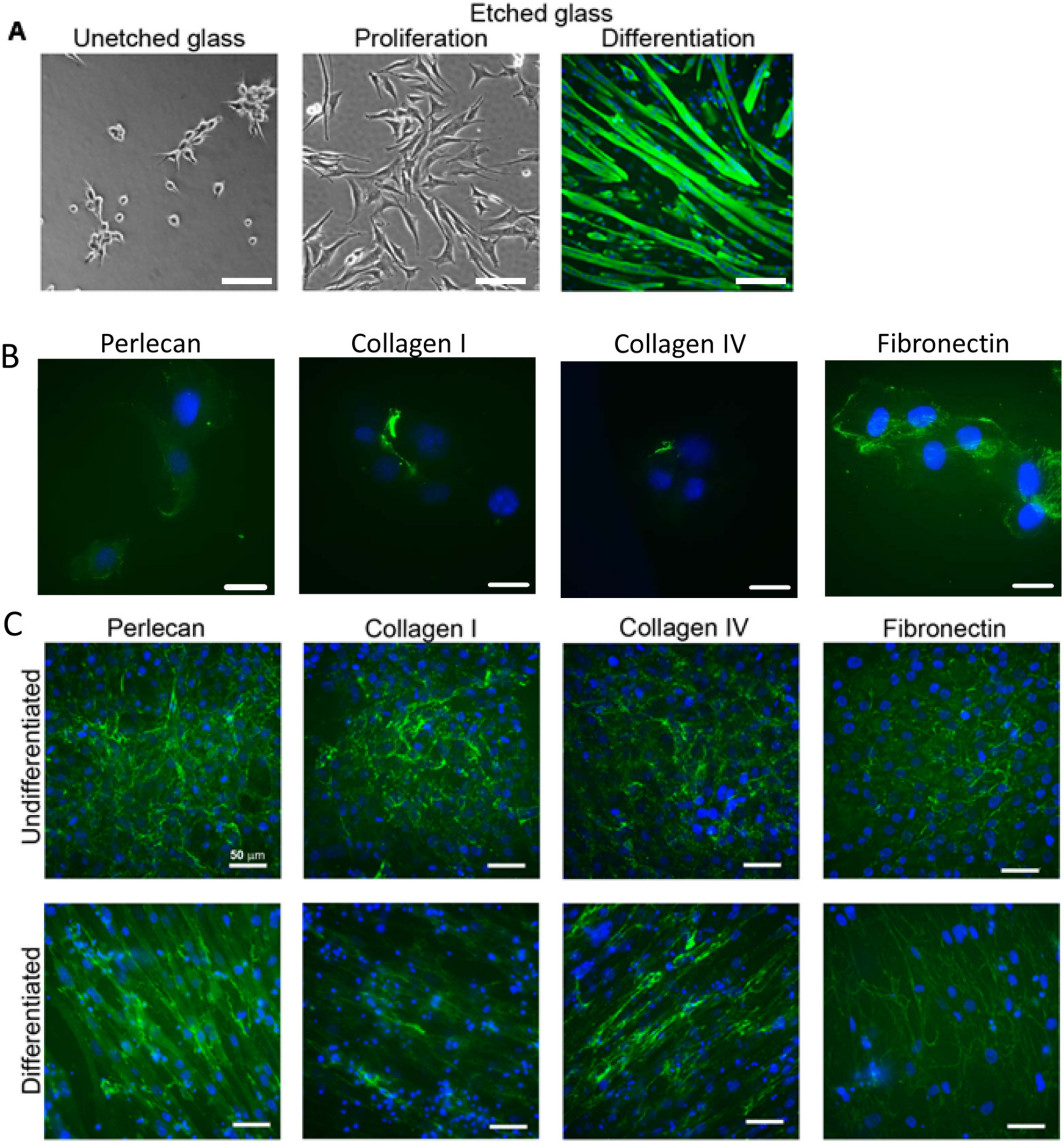
Secretion and orientation of matrix proteins by C2C12 cells under serum free conditions on etched glass. (a) C2C12 cells (3500/cm^2^) were seeded on un-etched and etched glass coverslips in the wells of a 12-well plate and cultured for 3 days in serum free proliferation medium. After 3 days, the media was changed to a serum free differentiation medium and cells were cultured for another 6 days. Post differentiation, cells were fixed and stained with anti-myosin mouse monoclonal (NOQ7.5.4D) antibody. The secondary antibody used was a goat anti-mouse alexafluor 488 antibody. Scale bar—100 μm. (b) The expression of matrix proteins by C2C12 myoblasts was examined on etched coverslips 3.5 hours after plating and in (c) undifferentiated (day 3 proliferation) and differentiated (day 6 differentiation) cell cultures. At these time-points cells were fixed and the coverslips immunostained using antibodies recognising perlecan, collagen I, collagen IV and fibronectin (all rabbit polyclonal antibodies.) The secondary antibody was goat anti-rabbit alexafluor 488 antibody. Nuclei are stained with DAPI (blue). Scale bar—50 μm.

As C2C12 cells failed to adhere in serum-free conditions on uncoated tissue culture plastic, it was investigated whether a non-protein surface that facilitated cell adhesion could support proliferation and differentiation. Etched glass coverslips were chosen as substrates for these experiments. C2C12 cells adhered, proliferated and differentiated on etched but not untreated glass coverslips in serum-free medium (Fig 9a). Immunostaining of differentiated cells with the MyHCB mAb revealed long multi-nucleate, striated myotubes (Fig 9a). Atomic force microscopy of etched and non-etched surfaces revealed the porous nanotopography of the etched surface (S2 Fig). To determine if these cells were secreting their own matrices, C2C12 cells in serum free culture were seeded onto etched glass coverslips for 3.5 hours before being fixed and immunostained using antibodies against collagen I, collagen IV, fibronectin and perlecan (Fig 9b). Extracellular fibronectin and perlecan were deposited at this very early time point suggesting these proteins may assist in cell adhesion to the etched glass, but only limited staining of collagen I and IV was evident. Matrix deposition over time was also investigated using serum-free cultures of C2C12 cells grown on etched glass coverslips for 3 days in proliferation media and 6 days in differentiation media. Proliferating cells deposited an organized ECM under the cell layer (Fig 9c). In differentiating cells, collagens I and IV were distributed around the length of the myotubes, perlecan was patchily distributed along myotube edges, whilst fibronectin displayed a filamentous web-like pattern (Fig 9c).

## Discussion

In this study, serum free cultures were used to examine myoblast proliferation and differentiation on solubilized and 3D decellularised quadriceps muscle matrices, where the matrices have been prepared by a method that removes cells but retains ECM components. In the serum free system C2C12 mouse myoblasts proliferated on solubilized acellular muscle matrix substrates, but progressed towards differentiation at a lower confluency than if they were cultured on collagen I or fibronectin substrates. Gene expression data suggested a similar differentiation pattern on muscle matrix and fibronectin, and the alignment of myosin expressing myotubes on muscle matrix and fibronectin were comparable. The 3D acellular muscle matrix supported cell growth and directed cell alignment, suggesting that *in vivo*, muscle ECM guides cell positioning. The serum free system used in this study ensured that undefined ECM molecules in the culture medium were not affecting cell behaviour, or modulating the contribution of the matrix protein substrates to myoblast differentiation. Interestingly, plating myoblasts on a non-protein substrate was also sufficient for adhesion, proliferation and differentiation without serum. Under these conditions, myoblasts secreted endogenous matrix proteins very early after plating and these proteins attached to the substrate and became well organised. Hence, provision of an exogenous matrix substrate is not essential for serum free cultures if matrix secretion is triggered and the culture surface supports the retention of secreted matrix proteins.

An adaptation of the method developed by Wang et al. (2011) to prepare an acellular liver matrix via perfusion [24] was found to be the best of the three methods examined for decellularising quadriceps muscle while preserving the ECM. The procedure consisted of sequential incubations of quadriceps in PLA_2_, sodium deoxycholate and DNase I. Although trypsin treatment effectively produced an acellular matrix, fibronectin was also removed, whereas SDS treatment did not remove DNA efficiently. In contrast, the use of PLA_2_ and DNase I to decellularize quadriceps muscle resulted in the effective removal of cellular material, as shown by DNA and cytoskeletal protein analyses (S1 Fig) while preserving the structure of the matrix (Fig 4). Although agarose gel electrophoresis and DAPI staining indicated a complete loss of DNA following PLA_2_ treatment, spectrophotometry indicated only an 82% reduction in nucleic acids in decellularised tissue. This may be due to differences in the sensitivities of the methodologies, as spectrophotometry is more sensitive than agarose gel electrophoresis and visualization of DAPI staining. Other studies have investigated the loss of nuclear material via haematoxylin and eosin (H&E) or DAPI staining [18–20], with similar results to our study. Gillies et al (2007) used a fluorescent DNA dye and showed a 95% reduction in nucleic acid content and no H&E staining following decellulatisation, yet western blotting still indicated that actin was present [20]. In contrast, we demonstrated a complete loss of the intracellular proteins myosin and β-tubulin. It is probable that some intracellular material remains after all decellularisation methods, but the amount detected depends on the method of measurement and the decellularisation method used.

Immunostaining indicated numerous ECM glycoproteins were retained, as antibody epitopes for collagens type I, III, IV and VI; fibronectin; laminin α2; proteoglycans perlecan, and a HS epitope EV3C3 were present (Figs 1 and 2). In some instances the staining intensity increased in the decellularised matrix (for example, the collagens), suggesting more antigenic sites were exposed following cell loss. Western blotting data supported the conclusion that the PLA_2_ decellularization method preserved many ECM proteins (Fig 3). Western blotting for collagens I and VI in the solubilized ECM extracts revealed intact α1 chains as well as high molecular weight collagen trimers. Evidence of intact laminin α2 chains, fibronectin monomers and perlecan were also obtained from the Western blotting analyses, although there did appear to be some reduction in fibronectin and perlecan. These data are consistent with those of Wang et al (2011), who demonstrated preservation of HS chains and perlecan in addition to collagens and fibronectin in decellularised liver prepared using PLA_2_ perfusion [24]. They suggested the retention of collagens enabled the preservation of collagen-binding molecules like laminins, fibronectin, and proteoglycans, and as a consequence, the cytokines and growth factors that bind these glycoproteins [24].

Overall, the PLA_2_ decellularisation protocol removed a high proportion of intracellular material, while leaving most ECM proteins intact. Although DeQuach et al (2010) describe an SDS-based decellularisation method that demonstrated good retention of matrix components [19] we found a large amount of residual nuclear material using this approach (S1 Fig). In contrast, trypsin treatment (as used by Stern et al (2009)) [18] showed excellent removal of nuclear material but it also stripped away a number of matrix proteins. An alternative method was described by Gillies et al (2011) [20]. They used osmotic shock to lyse skeletal muscle cells followed by latrunculin B and high salt treatment to removed actin and myosin, and DNase treatment to remove nucleic acid. This method showed excellent removal of DNA, but retention of actin and myosin protein fragments, and although the collagen content was consistent with that of intact muscle, the GAG content was reduced by approximately half [20]. This method retained the tissue architecture as scanning electron microscopy of their acellular tibialis anterior muscle revealed hollow tubular extracellular matrix structures [20], resembling the structure of our acellular quadriceps muscle revealed by the same technique.

To assess the functional attributes of our acellular quadriceps muscle matrix, the ability of the matrix to support the proliferation and differentiation of a murine myoblast cell line, C2C12 under serum free conditions was examined in two and three dimensional culture conditions. Two groups have reported the growth and differentiation of C2C12 myoblasts on solubilized skeletal muscle ECM [18, 19]. They reported enhanced proliferation and differentiation (increased myotube size and more nuclei per myofibre) in cells cultured on decellularised muscle matrix compared to collagen I. However, these studies used medium supplemented with fetal bovine serum for proliferation and horse serum for differentiation. As serum contains an ill-defined mixture of growth factors and soluble ECM matrix proteins [29], sera is likely to have introduced confounding factors. The serum free medium used here for C2C12 cell culture enabled a comparison of solubilized muscle matrix substrates with those of purified collagen I and fibronectin without the confounding variables introduced by serum. Purified collagen I was used for comparison, as it is a major component of skeletal muscle ECM. Fibronectin was used as a positive control because our previous unpublished data indicated that fibronectin was superior to collagen I for promoting C2C12 myoblast proliferation and differentiation in serum free media.

Under serum free conditions C2C12 myoblasts adhered to, and maintained their characteristic stellate shape, on fibronectin, collagen I and solubilised muscle matrix substrates (Fig 5). However, C2C12 myoblast proliferation was slower on muscle matrix and cell numbers plateaued earlier than on collagen I or fibronectin, such that the cell number on the pure protein substrates was approximately 1.5 times that on muscle matrix. This is in contrast to Stern et al (2009) who reported increased proliferation of C2C12 myoblasts on muscle matrix compared to collagen, albeit in the presence of serum. However, this effect was only seen at relatively high concentrations of matrix and collagen (0.2–0.5 mg/ml), whereas at lower concentrations (0.02–0.05 mg/ml; more similar to that used in our study) there were only small differences in proliferation rate between the different matrices [18]. It is possible the slower growth and earlier plateau in cell number on the muscle matrix substrate may be due to adult, muscle-specific factors in the matrix directing cell differentiation, even though the growth factors in the medium were designed to stimulate proliferation. However, it is important to note that we did not compare C2C12 myoblast behaviour on matrices decellularised by the methods used by other authors, and thus it is possible that the discrepancies between our results and others are due to the serum free culture rather than the retention of additional ECM components. De Quach et al (2010) also reported earlier differentiation of C2C12 myoblasts on muscle matrix compared to purified collagen and concluded that ECM components other than collagen in the muscle matrix retained biological activity and influenced C2C12 myoblast differentiation [19].

Slower proliferation may not be a disadvantage, as it allows time for the cells to organise and align prior to differentiation. For example, Ricotti et al (2012) evaluated the proliferation and differentiation of two myoblast cell lines on substrates of electrospun poly (hydroxybutyrate) (PHB) sub-micron fibers either highly oriented or randomly aligned, or films of PHB. The myoblasts proliferated more slowly when grown on oriented nanofibres compared to randomly aligned nanofibres or PHB films, but differentiated to form longer myotubes expressing higher levels of the genes characteristic of a differentiated phenotype [30]. In contrast, Tierney et al (2014) found that enhanced proliferation of satellite cells lead to larger regenerating muscle fibres *in vivo* [31]. They injected an inhibitor of Stat3 (a transcription factor required for myogenic differentiation) into regenerating muscle in a murine model. This led to increased satellite cell proliferation at 3 days, returning to control proliferation levels after 5 days. An additional effect of the inhibitor was faster repair and a larger cross sectional area of treated myofibers compared to those of non-treated controls at day 5, although this difference was not evident at day 25 [31]. These data suggest that transient rapid proliferation may be beneficial to regeneration. However, we found the muscle matrix appeared to regulate proliferation rather more than the pure matrix proteins, as on these latter substrates rapid proliferation progressed for longer, giving rise to very crowded cultures prior to differentiation and myotube formation.

In our study, C2C12 myoblasts fused to form mature myoblasts expressing MyHCB by day 4 of differentiation on collagen, fibronectin and muscle matrix. There were no significant differences in the thickness or number of nuclei per fibre on each substrate. These findings differ from those of DeQuach et al (2010), who reported that both myotube width and the number of nuclei per myotube significantly increased when myoblasts were grown on skeletal muscle matrix compared to collagen coated surfaces [19]. DeQuach et al (2010) used a higher concentration of muscle matrix than used in this study. However, in our system, surfaces coated with the higher concentrations of soluble matrix used by DeQuach et al (2010) and Stern et al (2009) were detrimental to C2C12 growth, differentiation and viability; as the consistency of the higher protein coating impeded cell migration and cell-cell contact (data not shown).

We found the most striking difference in myotube formation was that myotubes on collagen I were randomly oriented with many branched fibres, whereas myotubes on fibronectin and muscle matrix were aligned in parallel with less branching. Alignment of myotubes is a pre-requisite for functioning muscle. Others have described C2C12 cells cultured on fibronectin substrates as elongating, aligning and fusing earlier than cells cultured on laminin and gelatin coated substrates [32]. Vaz et al (2012) performed time-lapse imaging of C2C12 cells cultured on fibronectin and gelatin coated surfaces and found that C2C12 cells on fibronectin coated surfaces migrated further and with ‘directionality’, whereas cells on gelatin moved randomly [32]. Directional migration should assist cell alignment and hence myotube formation.

The expression of genes involved in differentiation from myoblasts to myotubes was investigated. Myogenic regulatory factors are transcription regulators that control muscle differentiation. *Myf5* is the first of these to be expressed during mouse embryogenesis and is a marker of committed satellite cells and myoblasts, whereas, *MYOG* triggers the expression of myotube specific genes [14]. In this study, *Myf5* was expressed at differentiation day 1 (Fig 7), and expression remained stable in myoblasts on collagen I, but decreased by day 8 in myoblasts on muscle-matrix and fibronectin. Brown et al (2012) found that *Myf5* decreased in C2C12 cells during differentiation, in contrast *MYOG* levels increased initially but decreased as the cells fully differentiated [33]. This is similar to our findings with C2C12 cells on fibronectin (Fig 7a and 7b). Another study measured mRNA levels of *Myf5* and *MYOG* in primary porcine muscle stem cells cultured on collagen I, laminin, fibronectin, gelatin and Matrigel [34]. In this case the expression of *Myf5* decreased with cell differentiation regardless of substrate; but *MYOG* expression in cells on Matrigel increased in a time dependent manner and was higher than in cells cultured on the other substrates at day 5 of differentiation [34]. On muscle matrix we found *MYOG* expression in C2C12 myoblasts increased as differentiation progressed (Fig 7c). Like solubilised muscle matrix, Matrigel is a complex mix of ECM proteins [35]. Stern et al (2009) measured the levels of myogenin protein at day 1 and 3 of differentiation on plastic and ECM coated surfaces and found that myogenin levels decreased while MyHC levels increased as the cells differentiated, and myogenin levels were higher in C2C12 cells cultured on skeletal muscle matrix surfaces than on uncoated surfaces [18]. This mirrors our mRNA data. Interestingly *MYOG* null (myoG^-/-^) myoblasts, that cannot differentiate *in vivo*, form myotubes as efficiently as wild-type myoblasts when cultured on gelatin [36]. Possibly, the *in vitro* requirements for myogenin are less stringent than *in vivo*, and factors in complex matrices maintain expression of this protein further into the differentiation pathway than is usually seen *in vitro*.

Myosin, the muscle contractile protein, consists of four light chains and two heavy chains. Myosin heavy chain has different isoforms: *MYH3* is an embryonic form whereas *MYH1* and *MYH7* are adult isoforms; *MYH1* is expressed in fast-twitch fibres and *MYH7* in slow-twitch muscle fibres [37, 38]. Cardiac actin (*ACTC1*) is expressed early during differentiation of skeletal muscle, but is down regulated as skeletal muscle alpha actin (*ACTA1*) is expressed [39]. *ACTA1* is the major actin isoform in muscle and is a late differentiation marker [37]. The mRNA levels for *MYH3* and *ACTC1* increased at day 4 of differentiation on all three surfaces, but decreased by day 8; with the drop in expression more pronounced on muscle matrix and fibronectin than on collagen I. It is thought *Myf5* initiates the expression of *MYH3* and *MyoD* enhances *MYH3* expression. *MyoD* is expressed slightly later than *Myf5*, appearing in myoblasts and myocytes, but decreasing markedly in myotubes [40]. It might be expected that a decrease in *Myf5* expression will be followed a day or two later by a corresponding decrease in *MYH3* expression. This was observed in a study of C2C12 cells [33]. Our data do not allow such a detailed interpretation, but the expression pattern of *Myf5* and *MYH3* in cells grown on fibronectin and muscle matrix are consistent with this report [33]. An early peak in *MYH3* expression was followed by a decline in human muscle myoblasts cultured on gelatin [37], which resembles our data for cells on collagen I. The decrease in *ACTC1* expression levels by day 8 on all three surfaces is probably associated with an increased expression of other actin isoforms, a conclusion that is supported by the expression pattern observed for *ACTA1* on all substrates (Fig 7).

Myotube maturation was indicated by the time dependent increase in expression of adult myosin (*MYH1* and *MYH7*) and skeletal muscle alpha actin (*ACTA1*) genes. The increase in *MYH1* expression at day 4 was greater on muscle matrix than on collagen I or fibronectin, suggesting myotubes on muscle matrix differentiated more quickly. Like the study by Brown *et al* (2012) of the expression of myosin heavy chain isoforms during C2C12 cell differentiation [33], our data showed a temporal pattern of gene expression. The expression levels of *MYH3* and *ACTC1* peaked early and then declined whereas *MYH1*, *MYH7* and *ACTA1* were expressed either at increasing levels at day 8 or at the same level as day 4. A difference between our data and theirs was in the expression levels of *MYH7*; they observed similar declines in *MYH7* and *MYH3* expression over the same period, and grouped *MYH7* with the “early” expressing isoforms [33]. From our data, grouping *MYH7* with adult isoforms is appropriate and fits better with the *in vivo* expression patterns of the myosin heavy chain genes. It is possible that culturing C2C12 cells on matrix proteins directs gene expression to fit more closely with what occurs *in vivo*.

Solubilization of ECM destroys positional information provided to cells by the location of molecules in an intact matrix. When C2C12 cells were seeded on acellular 3D matrices in serum-free medium (Fig 8) they lined up longitudinally, or organised into circular patterns, depending on the alignment of the endomysial matrix “tubes” that surround the muscle fibres. Thus, particular matrix proteins provided cues to direct myoblast adhesion and migration to specific locations on the acellular matrices (Fig 8). This should be considered when using muscle grafts for repair. A recent study described the lack of full functional recovery of volumetric muscle injuries in rats treated with muscle autografts and suggested this was because the grafts were not aligned in accordance with the muscle fibres in the injury bed [41]. Given our data that myoblasts align in certain ways according to matrix signals, if autografts are incorrectly orientated then cell alignment on the graft and the muscle will not match and will hinder regeneration of a contracting muscle. Others have used acellular muscle matrix as grafts and describe the population of these matrices by host cells that differentiate into multinucleated myofibres [42]. Blood vessels and myofibres have been described as penetrating further into the acellular matrix graft as time progressed [43]. We likewise saw myoblast migration into the acellular matrices and evidence of differentiation in our *ex vivo* cultures.

To investigate whether matrix protein surfaces are a requirement for myoblast growth and differentiation in serum free conditions, a non-protein surface was tested. Glass coverslips etched with a sodium hydroxide/ethanol solution were used as a substrate because these surfaces readily adsorb amino acid polymers and proteins from aqueous solutions [44]. The myoblasts adhered, proliferated and differentiated on etched but not on un-etched glass coverslips. Cells generally do not interact with a surface directly, but rather via proteins absorbed onto the surface [45]. In our serum free conditions, immuno-fluorescence experiments revealed fibronectin and perlecan were deposited on the substrate by the cells within 3.5 h of plating. After 3 days of proliferation, well-organized matrix substrates of collagens type I and IV, fibronectin and perlecan were evident below the cell layer. Thus, the etched surfaces adsorbed matrix proteins secreted from the myoblasts to mediate the first steps of cell adhesion, and as the cells proliferated the deposited fibronectin and type IV collagen were organized into fibril networks. These data suggest that scaffolds for muscle repair may not be restricted to matrix proteins (e.g. collagen), and materials that trigger matrix protein secretion and adsorb these secreted proteins could be an alternative.

We report here the use of a serum free culture system to examine myoblast proliferation and differentiation on solubilized and 3D muscle matrices and on a non-protein surface. We found that PLA_2_ treatment produced decellularised skeletal muscle retaining all the matrix proteins tested. The gene expression data and the alignment of MyHCB positive myotubes indicated that two-dimensional substrates of acellular muscle matrix may be superior to substrates of type I collagen, but comparable to fibronectin for supporting C2C12 differentiation. Furthermore, data from the 3D acellular muscle matrices indicate that particular “tracks” within natural muscle matrices favour myoblast attachment and alignment over other regions. This means the topography of the matrix directs the location of myotube formation and *in vivo* probably facilitates the organization of myotubes into functioning muscle fibres in particular locations. In addition to the physical and chemical cues myoblasts receive from the surrounding matrix, they also rapidly secrete their own endogenous matrix proteins such as fibronectin, collagens I and IV and perlecan. That is, myoblasts interact with the existing matrix or surface, but also secrete their own matrix to create a localized microenvironment, which may contribute to myotube formation. Clearly, muscle ECM is more than the sum of its individual components and soluble muscle matrix substrates, or pure matrix proteins, are not substitutes for an intact 3D muscle matrix.

## Supporting Information

S1 Fig Efficacy of decellularization processes as revealed by loss of DNA and cytoskeletal proteins from skeletal muscle sections. (A) DAPI staining of 10 μm control (a–c) and decellularised muscle sections (d–f) 10 μm sections. Sections were treated with trypsin (d), SDS (e) or PLA_2_ (f), scale bar—100 μm. (B) Agarose gels showing genomic DNA isolated from equal quantities of untreated muscle and muscle decellularised using trypsin, SDS or PLA_2_. Lane 1—1Kb Plus DNA marker, Lane 2—control muscle, lane 3—decellularised muscle. (C) Untreated and PLA_2_ treated muscle extracts were resolved on 4–15% Mini-PROTEAN TGX gradient gels and probed using antibodies against intracellular proteins myosin and β-tubulin. C—untreated muscle extract, PLA_2_—decellularised muscle extract.
(TIF)

S2 Fig AFM imaging of un-etched and etched glass. Glass coverslips (13 mm x 13 mm) were etched using NaOH and ethanol, washed and dried under a stream of nitrogen. AFM imaging was performed using Nanoscope III a atomic force microscope with a silicon nitride tip in contact mode and a long range scanner. The above images are amplitude images with a scan size of 5 x 5 μm^2^ and scale bar—2μm. Clean coverslips washed with ethanol were used as control glass.
(TIFF)
