# Supplementary figures and images for: Interactions between Skeletal Muscle Myoblasts and their Extracellular Matrix Revealed by a Serum Free Culture System
Source: PLoS One. 2015 Jun 1;10(6):e0127675. doi: 10.1371/journal.pone.0127675 (PMC4450880; doi:10.1371/journal.pone.0127675)

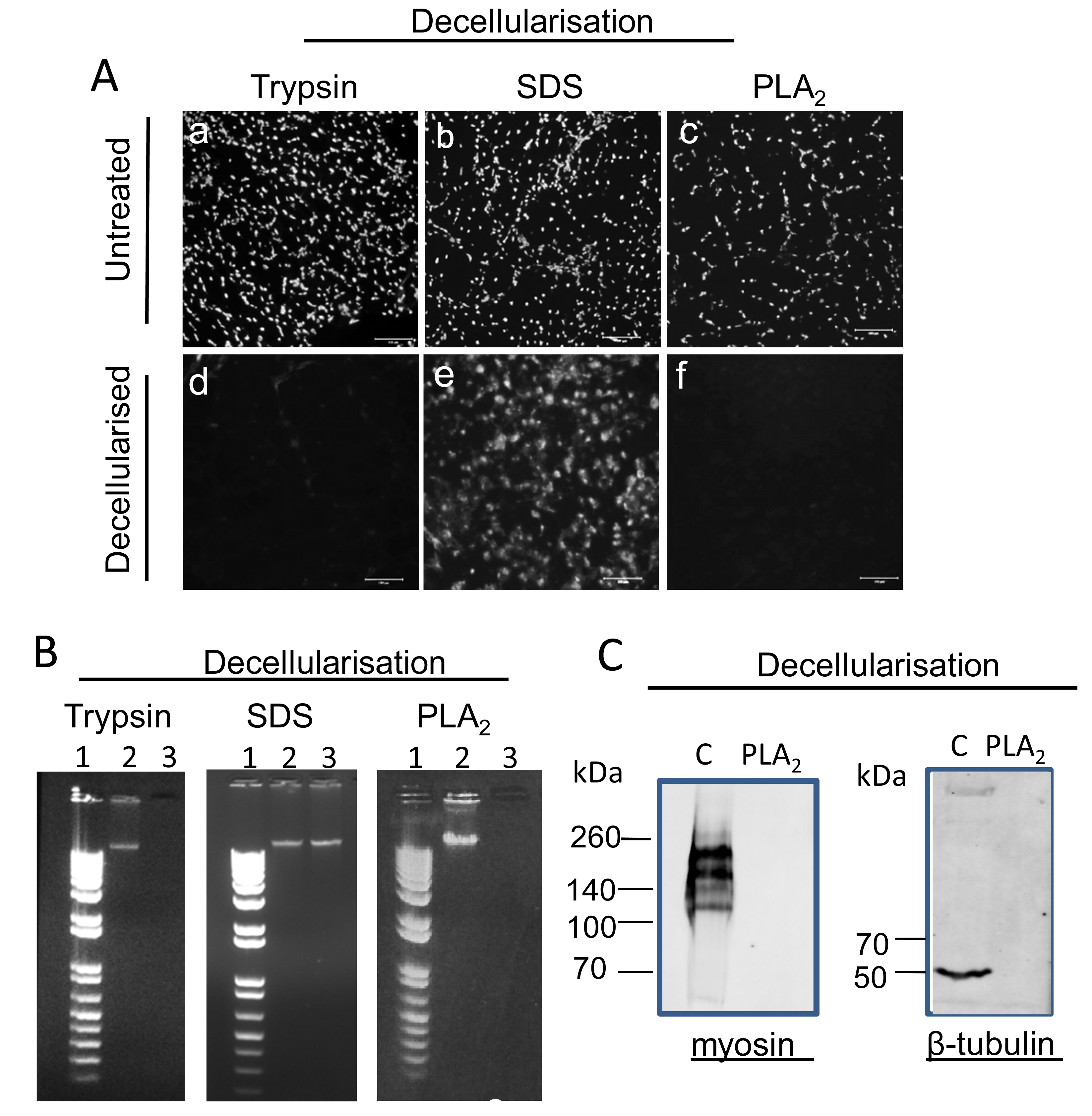

Supplement: S1 Fig — (A) DAPI staining of 10 μm control (a–c) and decellularised muscle sections (d–f) 10 μm sections. Sections were treated with trypsin (d), SDS (e) or PLA2 (f), scale bar—100 μm. (B) Agarose gels showing genomic DNA isolated from equal quantities of untreated muscle and muscle decellularised using trypsin, SDS or PLA2. Lane 1—1Kb Plus DNA marker, Lane 2—control muscle, lane 3—decellularised muscle. (C) Untreated and PLA2 treated muscle extracts were resolved on 4–15% Mini-PROTEAN TGX gradient gels and probed using antibodies against intracellular proteins myosin and β-tubulin. C—untreated muscle extract, PLA2—decellularised muscle extract. (TIF) [file pone.0127675.s001.tif]

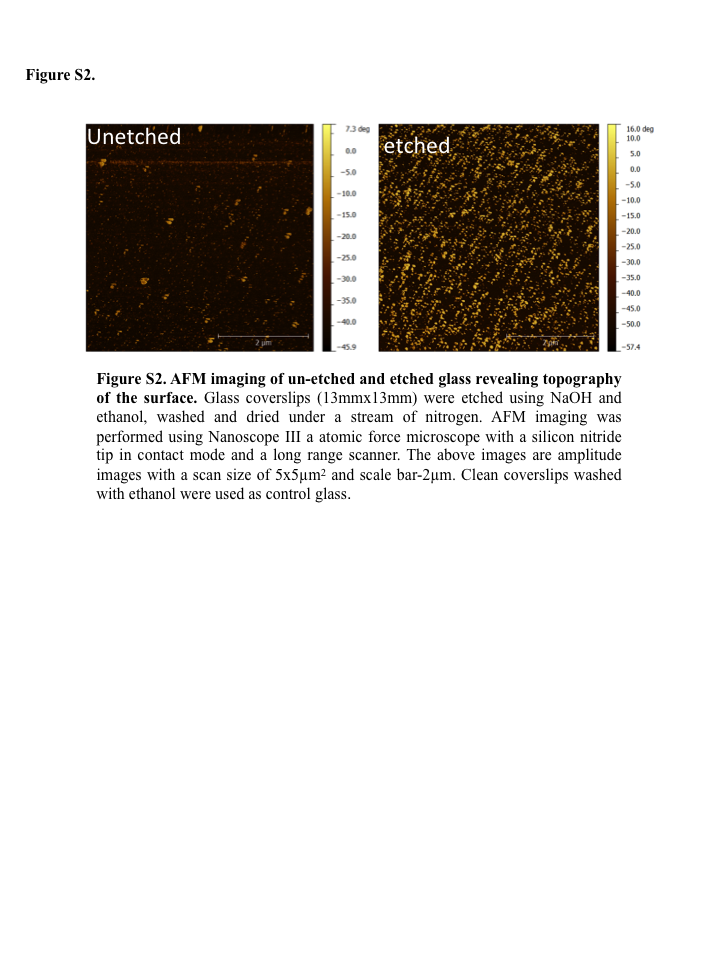

Supplement: S2 Fig — Glass coverslips (13 mm x 13 mm) were etched using NaOH and ethanol, washed and dried under a stream of nitrogen. AFM imaging was performed using Nanoscope III a atomic force microscope with a silicon nitride tip in contact mode and a long range scanner. The above images are amplitude images with a scan size of 5 x 5 μm2 and scale bar—2μm. Clean coverslips washed with ethanol were used as control glass. (TIFF) [file pone.0127675.s002.tiff]
